# Serum levels and biochemical characteristics of human ovarian carcinoma-associated antigen defined by murine monoclonal antibody, CF511.

**DOI:** 10.1038/bjc.1989.397

**Published:** 1989-12

**Authors:** K. Ohkawa, Y. Tsukada, M. Murae, E. Kimura, K. Takada, T. Abe, Y. Terashima, K. Mitani

**Affiliations:** Department of Biochemistry, Jikei University School of Medicine, Tokyo, Japan.

## Abstract

**Images:**


Br. J. Cancer (1989), 60, 953–960 © The Macmillan Press Ltd., 1989

### Serum levels and biochemical characteristics of human ovarian carcinoma-associated antigen defined by murine monoclonal antibody, CF511

K. Ohkawa[1], Y. Tsukada[2], M. Murae[3], E. Kimura[3], K. Takada[1], T. Abe[1], Y. Terashima[3] & K. Mitani[4]

[1]*Department of Biochemistry, Jikei University School of Medicine, 3-25-8, Nishi-Shinbashi, Minato-ku, Tokyo, Japan;* [2]*Department of Biomedical Research, Special Reference Laboratory Inc., Tokyo, Japan;* [3]*Department of Obstetrics and Gynaecology, Jikei University School of Medicine, Tokyo, Japan, and* [4]*Biochemical Research Laboratory, Tokuyama Soda Inc., Fujisawa, Japan.*

**Summary** The murine monoclonal antibody (Mab) against human common epithelial ovarian carcinoma, CF511, was generated by immunising mice with human fetal tissue extract from early first trimester, followed by booster injection of an ovarian cancer cell line. Mab CF511 recognised the 600 kDa sialylated glycoprotein as different from previously known tumour associated-marker antigens. Distribution of the Mab CF511-recognised antigen (CF511 antigen) in various tissues and sera was investigated. In immunohistochemical analysis, Mab CF511 reacted strongly with tumour cells of ovarian serous, clear cell, endometrioid and undifferentiated carcinoma and partially with those of mucinous carcinoma. Mab CF511 also reacted with breast carcinoma as well as lung carcinoma. In normal tissues, Mab CF511 cross-reacted with only five tissues, namely lung, breast, thyroid gland, fallopian tube and uterus. Serum levels of CF511 antigen were tested by ELISA inhibition using Mab CF511. This assay showed the circulating CF511 antigen levels to be elevated in 25 of 36 sera from patients with various clinical stages of common epithelial ovarian carcinoma compared to three of 47 and three of 111 sera from patients with other benign gynaecological diseases, including ovarian cysts, uterine fibroids with or without endometriosis and normal healthy subjects, respectively. For the relation between antigen levels and clinical stages of common epithelial ovarian carcinoma, greater than 34.0% ELISA inhibition was detected in 100% of patients with advanced stages (FIGO III and IV) compared with in 35.3% with early stages (FIGO I and II) patients. While patients with breast carcinoma (100%) and lung carcinoma (100%) also had elevated circulating CF511 antigen levels, patients with hepatoma, colorectal carcinoma and gastric carcinoma had no detectable elevation of antigen. Although the test showed a high degree of specificity, the detection of an elevated CF511 antigen level would not be so helpful in distinguishing patients with ovarian carcinoma from those with either breast carcinoma or lung carcinoma. These data suggest that CF511 antigen is a useful new ovarian tumour marker for diagnosis and management of the disease.

Despite recent advances in the therapy of ovarian malignancy, ovarian carcinoma is the most lethal of all gynaecological carcinomas. Because of an insidious disease arising from a deep-seated organ, initial detection is often delayed. The common epithelial ovarian carcinomas constitute the vast majority of all ovarian cancers (Petterson, 1985).

A large number of monoclonal antibodies (Mabs) against ovarian carcinoma have been produced using hybridoma technology, mainly by immunising mice with cancer cell lines or their extracts, in an attempt to identify tumour-specific or tissue-specific markers (Sakakibara *et al.*, 1988; Nakagawa *et al.*, 1987; Miotti *et al.*, 1985, 1987; Mattes *et al.*, 1984, 1987; Tsuji *et al.*, 1985; Tagliabue *et al.*, 1985; Croghan *et al.*, 1984; Bhattacharya *et al.*, 1982; Bast *et al.*, 1981). As yet, however, none of the antigens identified appears to be tumour-specific. The expression of ovarian and other cancer associated antigen(s) recognised by Mab(s) has been frequently detectable in normal tissues, especially in fetal tissues, by further extensive screening procedures. In this sense, some cancer associated antigens have been considered to be onco-developmental and/or tissue differentiation antigens (Feizi, 1985; O'Brien *et al.*, 1986).

Therefore, we have taken an alternative approach, i.e. immunised mice with embryonal tissues from fetus in early first trimester, given booster injections of an ovarian cancer cell line. The aim was to obtain efficiently antibodies which could react to both carcinoma and fetal cells. In this study we report one Mab, CF511, obtained using this novel approach, some biological properties of the antigen (CF511 antigen) and percentage incidence of the CF511 antigen in sera from ovarian carcinoma patients.

Correspondence: K. Ohkawa.
Received 20 February 1989; and in revised form 2 June 1989.

### Materials and methods

### *Human tissues*

Fresh normal and neoplastic adult tissues were obtained either at the time of surgery or at autopsy. Fetal tissues from around the tenth week of gestation were obtained during a therapeutic abortion in accordance with the guidelines (Ethicality of the limits to the use of organs from dead fetuses and neonates in research) of the Japanese Society of Obstetrics and Gynaecology.

### *Cell line*

The human ovarian clear cell carcinoma cell line, HAC-2, which was provided by Dr M. Nishita, Tsukuba University school of Medicine, was maintained *in vitro* by conventional methods. Recently an HAC-2 cell clone was able to replicate continuously but slowly in a chemically defined, serum-free medium supplemented with $Na_2SeO_3$ (Nakabayashi *et al.*, 1982). No CA125 antigen was produced by HAC-2 cells under either set of culture conditions as determined by radioimmunoassay.

### *Cell and tissue extracts*

Solid tissues were minced separately with scissors into small fragments and washed twice with 0.15 M NaCl solution. The tissue fragments were then placed into 10 volumes of ice-cold extraction buffer (10 mM Tris-HCl, pH 7.2, 0.15 M NaCl, 0.02% $NaN_3$, 1 mM phenylmethylsulphonyl fluoride and 0.5% Nonidet P 40) and homogenised with a Polytron homogeniser for 3 min on ice. Confluent monolayers of cultured cells were detacted by 0.02% EDTA in Dulbecco PBS(–), washed and resuspended in 10 volumes of ice-cold extraction buffer. The homogenates and cell suspension were then sonicated for 1 min, incubated for 20 min on ice, and centrifuged at 23,000 g for 20 min at 0°C. The supernatants were kept frozen at − 80°C until use. The protein concentrations of the extracts were determined by the method of Lowry et al. (1951).

*Production of Mabs*

BALB/c male mice were immunised 4-weekly with intraperitoneal (i.p.) injections of 2 mg protein of fetal extract emulsified in Freund's complete adjuvant. The extract was prepared from the homogenate of fetal tissues (excised liver and small intestine). Four weeks later the mice were given booster i.p. injections of $5 \times 10^6$ HAC-2 cells. Three days following the booster, the spleen cells from the immunised mice were fused by 50% polyethylene glycol (Boehringer Mannheim, FR Germany) with P3X63Ag8U-1 myeloma cells. Resulting hybrids were selected in HAT medium. Supernatants were initially screened for reactivity against HAC-2 cells using cell-target enzyme-linked immunosorbent assay (cell-ELISA). Target cell-coated plates were prepared as follows. HAC-2 cells, $2-3 \times 10^4$ per well, were cultured in tissue culture microplates (Corning no. 25860, USA) for 2–3 days to obtain a monolayer. Wells were gently washed once with PBS and then fixed with 0.05% glutaraldehyde (Sigma, USA) in PBS for 5 min at room temperature. For the saturation of free aldehyde groups they were incubated for 30 min with 1% bovine serum albumin (BSA, Sigma, USA) in 0.1 M glycine in PBS. Wells were washed twice with PBS and plates were incubated for 20 min with 0.3% (wt/vol) $H_2O_2$ in methanol for blocking the activity of endogenous peroxidase. Wells were blocked with Tris-buffered saline (TBS) containing 5% skimmed milk (Difco, USA) and 0.1% merthiolate for 2 h at room temperature or overnight at 4°C. Plates were washed twice with TBS containing 0.05% Tween 20 (T-TBS), and 50 µl of hybridoma culture supernatants were added and incubated for 4 h at room temperature. The wells were washed three times with T-TBS, followed by addition of 50 µl horseradish peroxidase (HRP) labelled goat anti-mouse immunoglobulin (Ig) conjugate (Cappel, USA) diluted 1/1,000 in 5% skimmed milk in TBS and further incubated for 2 h at room temperature. After repeated five times washing, 150 µl of *O*-phenylenediamine as a substrate was added and measured absorbence at 492 nm was determined after 20 min of colour development with a microplate reader (MPR A4 Toyo Soda, Japan). The hybridomas cloned by limiting dilution, expanded *in vitro* and were grown as an ascitic form in pristane-primed BALB/c mice. The spent culture media and ascites fluid were used as the source of antibody.

*Immunoperoxidase staining*

Formalin fixed and paraffin embedded materials of common epithelial ovarian carcinomas and normal tissues were dewaxed, rehydrated through graded alcohol and blocked endogenous peroxidase by the conventional method by immersion in 0.3% $H_2O_2$ in methanol for 20 min. After washing in PBS, followed by treatment with normal horse serum, either hybridoma supernatants or Mab (10 µg $ml^{-1}$) was incubated on each section for 60 min. The biotinylated anti-mouse Ig horse antibody (Vector, USA) followed by avidin-biotinylated HRP (Vector, USA) was applied for 30 min. Sections were incubated for a few minutes in peroxidase substrate (3,3′ diaminobenzidine 4 HCl, DAB) solution. After being washed with distilled water, the sections were counter-stained with haematoxylin.

*Sodium dodecyl sulphate-polyacrylamide gel electrophoresis (SDSPAGE) and immunoblotting*

SDSPAGE was carried out by the discontinuous system of Laemmli, using 7.5% acrylamide gels under reducing conditions. Transfer of antigens to nitrocellulose paper was performed according to the method of Towbin et al. (1979). Immunoblotting analysis was performed with residual binding sites which were blocked by incubation of blotted nitrocellulose paper with 5% skimmed milk in TBS, incubated with Mab CF511 culture supernatant at 4°C overnight, followed by incubation with properly HRP-conjugated anti-mouse Ig goat antibody (Cappel, USA) for 2 h at room temperature. The peroxidase activity was detected by exposing the membrane to 0.2 mg $ml^{-1}$ of DAB, 0.01% $H_2O_2$ in TBS for 10 min.

*Gel filtration*

HPLC system (TSK gel G-3000SW, 7.5 mm × 60 cm, Toyo Soda, Japan) was used. The column was equilibrated with 50 mM PB, pH 6.8, 0.25 M NaCl, at a flow rate of 0.43 ml $min^{-1}$. The antigenic content in each fraction was assayed by ELISA inhibition test.

*ELISA for binding assay (antigen coated ELISA)*

In order to confirm the releasing ability of Mab-recognised antigen to the medium, wells of the 96-well microtitre plate (Immunoplate, Nunc, Denmark) were coated with 50 µl of spent culture medium from HAC-2 grown in serum free medium (100 µg $ml^{-1}$ protein, HACCM(−)), blocked with 5% skimmed milk in TBS for 1 h at room temperature and then the antibody solution, HRP-labelled antibody and the substrate were reacted for the cell-ELISA as described above.

*ELISA inhibition test*

Either spent culture medium of HAC-2 or sera were pre-incubated with an equal volume of purified antibody solution. Aliquots were inoculated into a microtitre well precoated with HACCM(−) for further incubation. To the wells on the microtitre plate was then added HRP-conjugated goat anti-mouse Ig (1/1,000 diluted with 5% skimmed milk-TBS) followed by determination at 492 nm by the addition of substrate as mentioned above. Results were expressed in terms of percentage of inhibition of ELISA signal according to the following equation: % inhibition of ELISA = [1 − (test OD − control OD)/(maximum OD − control OD)] × 100.

*Purification of Mab CF511*

Purification of Mab CF511 was performed by precipitation with 50% ammonium sulphate and gel filtration chromatography using Sephacryl S-300 (2.5 × 60 cm, buffered with TBS containing 0.02% $NaN_3$, Pharmacia, Sweden), followed by HPLC (TSK gel G-4000SW, 7.7 mm × 60 cm, buffered with 50 mM PB, pH 6.8, 0.25 M NaCl, Toyo Soda, Japan). Fractions determined by the Ouchterlony test to have reacted with goat anti-mouse IgM (Cappel, USA) were collected and used.

*Human sera*

Serum samples from patients were supplied by Jikei University hospital. Sera of healthy volunteers were obtained in our laboratories and from Tokuyama Soda laboratories. The samples were stored at − 80°C until use.

*Enzyme and reagent treatment*

All treatments for enzyme and reagent were performed in the Eppendorf microtubes because the treated and digested fragments released from the antigen molecule, with or without maintaining the antigenicity, had to be carefully determined. Each tube of 3 mg packed cells (approximately $5 \times 10^5$ cells), which were detached from the culture dish by 0.02% EDTA treatment, was treated with either enzymes or reagents. For NaOH or $NaIO_4$ treatment, packed cells were treated either with 0.1 N NaOH and 2 M $NaBH_4$ for 25 h at 37°C or 15 mM $NaIO_4$ (Sigma, USA) in 10 mM acetate buffer, pH 4.5, for

15 h at room temperature in dark. The cultures treated with neuraminidase (0.1 U $ml^{-1}$, Seikagaku Kogyo, Japan), 0.125% trypsin (Difco, USA) or proteinase K (1 mg $ml^{-1}$, Boehringer Mannheim, FR Germany) were centrifused and each supernatant and precipitate was subsequently diluted in SDS containing PAGE sample buffer and boiled for 5 min at 100°C. Resulting samples were analysed by SDSPAGE and immunoblotting as described above.

*Cross-reactivity of Mab CF511 to well known tumour markers*

To assess the immunological specificity of Mab CF511, cross-reactivity of the recognised tumour-associated markers to CF511 antigen were tested for dose–response inhibition against the immune reaction between Mab CF511 and CF511 antigen. The standard reagents included in the commercially available immunoassay kits were used as a source of CA 19-9, CA 125 and CA 15-3 (Centocor, USA), DU-PAN-2 (Kyowa Medix, Japan), SLX (Ohtsuka assay Lab, Japan) and CSLEX (SRL, Japan).

*Type of Ig*

The subclass of Mab was determined by the Ouchterlony test with rabbit IgG against different mouse Ig subclasses (ICN, UK).

### Results

*Establishment of the Mab CF511 and its reactivity*

The supernatant solutions from the 682 wells containing growing hybridomas were screened initially by cell-ELISA, followed by antigen (HACCM(–)) coated-ELISA and finally by immunohistochemical examination, and five hybridomas were established and cloned. Of the five Mabs obtained by this fusion, Mab CF511 (IgM, κ) reacted most strongly with tissue sections of ovarian carcinomas immunohistologically as well as in the ELISA test and further examinations were carried out. Results of an immunohistological screening study of the reactivity of Mab CF511 are presented in Tables I and II. Mab CF511 stained all common epithelial ovarian carcinomas tested, except four with mucinous histologies, with no apparent selectivity for either papillary or infiltrating medullary types. The immunoreaction of Mab CF511 occurred at the cell membrane. In tested normal adult tissues, Mab CF511 reacted only with five tissues: bronchial and alveolar epithelia in the lung, epithelial cells of the mammary gland, follicular lining cells of the thyroid gland, epithelial cells of fallopian tube and a few glandular cells of the uterine endometrium. The results obtained from immunohistological analysis coincided fully with the reactivities of the Mab CF511 with various tissue extracts using the method of immunoblot analysis. The level of CF511 antigen in fetal extract was extremely low and weak but a significant band was found at a level of more than 100 times concentration of the protein content in the extract (Figure 1).

*Levels of CF511 antigen in patients' sera*

ELISA inhibition test was initially carried out. First, quantitative absorption of Mab CF511 was performed. The appropriate dilution of Mab was established in titring the antibody against HACCM(–) using the antigen coated-ELISA system. The dilution used in the absorption test was 1:10 (5 μg $ml^{-1}$) and, by this test, the appropriate dilution of patients' sera was found to be 1:3 (Figure 2). The 1:10 diluted Mab CF511 was adapted to ELISA, and 1:3 diluted sera were treated for CF511 antigen levels by their ability to inhibit this ELISA signal according to the result of pre-incubation with the Mab CF511. Figure 3 shows the level of ELISA inhibition produced by a total of 219 sera from healthy volunteers, patients with common epithelial ovarian carcinoma, other carcinomas and benign diseases. The results are expressed as a percentage of inhibition of the ELISA signal obtained with a pooled normal human control sera. The cut-off level was based on the observed range of inhibition (mean + 2 s.d., 34.0%) of reactivities using sera from healthy individuals. Sixty-nine per cent (25/36) of sera from patients with common epithelial ovarian carcinoma showed a significant inhibition of ELISA signal compared to 0/2 sera from malignant ovarian tumour arising from germ cell or sex cord mesenchymal cell, 0/7 sera from benign ovarian tumour (including six cases of ovarian cyst), 3/40 sera from uterine fibroid (with or without endometriosis) and 3/111 sera from healthy individuals. The relation between the CF511 antigen levels and clinical stages was evaluated in 32 common epithelial ovarian carcinoma patients who were accurately diagnosed for clinical stage at operation. Values of more than 34.0% ELISA inhibition were detected in 6/17 cases (35.3%) with early stage (stage I, 16 cases; stage II, one case) and in 15/15 cases (100%) with advanced stage (stage III, 13 cases; stage IV, two cases), suggesting an association between antigen level and tumour burden. All sera from lung carcinoma and breast carcinoma patients also showed ELISA inhibition but no ELISA inhibition was noted in sera from patients with hepatoma or with gastric carcinoma (including one case of Krukenberg tumour) or colorectal carcinoma. Furthermore, the menstrual cycle and pregnancy had no effect on circulating antigen levels (data not shown).

**Table I** Immunohistochemical reactivity of Mab CF511 to neoplasms

| *Tissue* | *No. of specimens stained/total tested*[a] | *Staining intensity*[b] |
|---|---|---|
| Ovarian tumour | 27/35 | – |
| cystadenocarcinoma | 27/31 | – |
| serous | 6/6 | 3+ |
| mucinous | 4/8 | + |
| clear cell | 7/7 | 3+ |
| endometrioid | 6/6 | 2+ |
| undifferentiated | 4/4 | 3+ |
| granulosa theca cell tumour | 0/2 | – |
| yolk sac tumour | 0/2 | – |
| Lung carcinoma | 3/3 | 3+ |
| Breast carcinoma | 2/2 | 3+ |
| Colon carcinoma | 0/2 | – |
| Gastric carcinoma | 0/4 | – |
| Hepatoma | 0/3 | – |

[a]Specimens from unrelated donors. [b]Mean staining intensity on a scale of 0–3+: –, no reactivity; +, weak reactivity; 2+, moderate reactivity; 3+, strong reactivity.

**Table II** Immunohistochemical reactivity of Mab CF511 to normal tissues

| *Tissue* | *No. of specimens stained/total tested* | *Staining intensity* |
|---|---|---|
| Brain | 0/1 | – |
| Breast | 1/4 | +, epithelial cell |
| Oesophagus | 0/2 | – |
| Stomach | 0/2 | – |
| Lung | 4/4 | 3+, alveolar cell bronchial cell |
| Trachea | 0/4 | – |
| Small intestine | 0/2 | – |
| Kidney | 0/2 | – |
| Urinary bladder | 0/2 | – |
| Colon | 0/2 | – |
| Liver | 0/4 | – |
| Gall bladder | 0/2 | – |
| Pancreas | 0/2 | – |
| Muscle | 0/2 | – |
| Spleen | 0/2 | – |
| Thyroid gland | 3/3 | +, follicular cell |
| Uterus | 4/4 | 2+, glandular cell |
| Ovary | 0/4 | – |
| Fallopian tube | 2/4 | +, epithelial cell |

See Table I for key.

**Figure 1** SDSPAGE and immunoblot analysis of the antigen recognised by Mab CF511 (fetal extract (10 mg protein $ml^{-1}$) of **a**, cell or tissue extract (0.1 mg protein $ml^{-1}$); **b1** and **c3**, HAC-2 cells; **b2**, ovarian clear cellcarcinoma; **b3**, ovarian mucinous carcinoma; **b4**, ovarian serous carcinoma; **b5**, ovarian undifferentiated carcinoma; **b6**, hepatoma; **b7**, colon carcinoma; **b8**, gastric carcinoma; **c1**, thyroid gland; **c2**, lung; **c4**, kidney; **c5**, endometrium). (▷), origin; Mol. wt, molecular weight; BPB, bromophenol blue.

**Figure 2 a,** Titration of Mab CF511 against HACCM(−) as antigen. Serial 10-fold dilutions of hybridoma culture supernatant with (▲) or without (■) pre-incubation with HACCM(−) were tested in triplicate against antigen coated ELISA as described in Materials and methods. **b**, Quantitative absorption of Mab CF511 with two patients' sera with ovarian carcinoma (●, ■) and with sera from healthy volunteers (O, □). Suitable diluted Mab were absorbed with serially diluted sera and 1% BSA in TBS as a control. The residual antibody activity was determined in triplicate against HACCM(−) as antigen with antigen coated ELISA as described in Materials and methods. Residual antibody activity (% of control) = [OD (Mab absorbed by sera)]/[OD (Mab absorbed by 1% BSA in TBS)] × 100. For each assay, s.d. of the triplicate determinations were within the markers.

**Figure 3** Inhibition of Mab CF511 ELISA original by sera from patients with various cancerous and benign diseases as well as healthy volunteers. Positive/negative cut-off was determined by preliminary tests of sera from healthy volunteers to establish a normal range of inhibition as described in Materials and methods. Results were expressed in terms of % of inhibition of ELISA signal as the following equation: % inhibition of ELISA = [1 – (test OD – control OD)/(maximum OD – control OD)] × 100. Each point represents the mean of duplicate determinations of different two assays.

### *Biochemical properties of CF511 antigen*

The elution profile of gel filtration analysis of the antigen either in an extract of HAC-2 cells or in a HACCM(–) is shown in Figure 4. More than 90% of the applied activity was detected in fractions eluting just after the elution volume of thyroglobulin (660 kDa). Figure 1 shows immunoblot analysis of SDSPAGE at the reduced condition of various antigen preparations stained with Mab CF511. By immunoblot analysis using cell extracts from common epithelial ovarian carcinomas and from some normal tissues as well as from HAC-2 cells, Mab CF511 stained one band of apparent molecular weight in excess of 330 kDa, but Mab CF511 did not stain the thyroglobulin under the same conditions (data not shown).

Evidence that Mab CF511 reacted with a glycoprotein antigen was derived from six different experiments (Figure 5, Table III). Initially characterisation of the CF511 antigen was performed by neuraminidase, trypsin and proteinase K digestion. After enzyme digestions, as shown in Figure 5, the reactive bands to antibody were not found in the lanes of the

**Figure 4** HPLC-gel filtration profile of HACCM(–). Fractions were tested for absorbence at 280 nm for CF511 antigen by ELISA inhibition as described in Materials and methods. 1, blue dextran; 2, thyroglobulin; 3, amylase; 4, BSA; 5, carbonic anhydrase.

samples derived from supernatants but only in those from precipitates (cell lystates). The results show that the binding ability of Mab CF511 to the antigen was partially destroyed by the treatment with neuraminidase. This result indicated that the antigenic sites recognised by Mab CF511 would contain sialic acid residues (Figure 5). The same result was also demonstrated by antigen coated or cell-ELISA.

Pre-incubation of antigen for 1 h with neuraminidase resulted in a 55% decrease in ELISA signal (Table III). However, trypsin treatment considerably increased the binding capacity of the antibody to defined antigen, separated by SDSPAGE, transblotted on the nitrocellulose paper; however, migration of the antigen into the gel markedly broadened and resulting bands were separated into four (Figure 5). In contrast, treatment with proteinase K reduced the binding capacity of antibody to antigen on the nitrocellulose paper. However, weak but significant bands were noticeable. These findings suggested that some parts of the protein molecule might play a role in maintaining antigenicity. Since CF511 antigen was partially digested by trypsin without loss of the antigenicity, lysyl and arginyl bonds of peptide chains may not affect the antigenicity. The CF511 antigen was quite sensitive to 0.1 N NaOH and 2 M $NaBH_4$ (Figure 5). Moreover, treatment with proteinase K, 0.1 N NaOH and 2 M $NaBH_4$ failed to make detectably smaller molecular weight antigenic species. These findings may be because of inadequate transfer during the immunoblotting procedure of smaller fragments consisting mainly or entirely of carbohydrate. The CF511 antigen reactivity remained unchanged by heat treatment for 15 min at 100°C (Table III). By treatment with $NaIO_4$, the immunoblotting analysis showed that the detection of the antigen demonstrated that CF511 binding to the antigen was not sensitive but increasing by the treatment with $NaIO_4$; however, migration of the antigen into the gel decreased after this treatment, indicating that CF511 antigen contained carbohydrate chain and changes its conformation without decreasing antigenicity. After treatment with $NaIO_4$, the antibody binding activity to antigen was also increased more than 70% of control when assay was carried out by antigen coated or cell-ELISA (Table III). Since mild periodate oxidation at acidic pH has been shown to cleave carbohydrate vicinal hydroxyl groups without altering the structure of the polypeptide chains, the increased antibody binding upon periodate oxidation suggests that Mab CF511 may bind to the antigenic sites of peptide backbone rather than to that of carbohydrate molecules on the sialylated glycoprotein antigen.

### *Comparison of CF511 antigen with other tumour markers*

The reactivity of Mab CF511 was compared with those of Mab OC 125 (Bast *et al.*, 1981), DU-PAN-2 (Metzger *et al.*, 1984) and DF3 (Hayes *et al.*, 1985), which recognised the peptide antigen in part, the sialylated carbohydrate antigen, as well as NS 19-9 (Koprowski *et al.*, 1979), FH 6 (Fukushi *et al.*, 1984) and CSLEX1 (Fukushima *et al.*, 1984), which recognised the sialylated Lewis$^a$, the sialylated Lewis$^x$-i and sialylated Lewis$^x$, respectively. Mab CF511 (5, 2, 0.5 $\mu g\ ml^{-1}$)

**Table III** Effect of chemical or enzyme treatment of antigen on Mab CF511[a] binding activity

| *Agent* | *Residual antigen activity (%)* |
|---|---|
| Chemicals | |
| control | 100 |
| $NaIO_4$ (15 mM, room temp 15 h) | 170 |
| heat (100 C, 15 min) | 104 |
| Enzymes | |
| control | 100 |
| neuraminidase (0.1 U $ml^{-1}$, 37 C, 1 h) | 45 |

[a]Data obtained from antigen coated- or cell-ELISA determination.

**Figure 5** CF511 antigen (HAC-2 cell) was treated with either enzymes or chemical reagents. After treatment of packed cells with each reagent, resulting supernatants and precipitates were then subjected to SDSPAGE followed by immunoblotting with Mab CF511 as described in Materials and methods. **a**, lane 1, PBS; 2, 0.125% trypsin (supernatant); 3, 0.125% trypsin (cell lysate); 4, proteinase K (1 mg ml$^{-1}$, supernatant); 5, proteinase K (cell lysate); 6, NaOH/$NaBH_4$ (mixture of supernatant and cell lysate). **b**, Cells in 10 mM acetate buffer pH 4.5 with (2) or without (1) 15 mM $NaIO_4$. **c**, Cells in 10 mM acetate buffer, pH 6.5, 10 mM $CaCl_2$ with (2, 2 h; 3, 1 h) or without (1) 0.1 $\mu$ ml$^{-1}$ neuraminidase.

was pre-incubated for 17 h at 4°C with each standard substance contained in each assay kit. Then each incubation mixture was assayed with inhibition ELISA assay as well as according to the presented usual manner mentioned in each kit. As shown in Table IV, Mab CF511 did not react with the standard substance of each assay kit and no remarkable competitive inhibition to make the standard curve for each assay system was observed (data not shown).

### Discussion

Mab CF511 was raised by immunising mice with a tissue extract from 9–10-week-old fetus. The antibody binds to a 600 kDa glycoprotein antigen found in many common epithelial ovarian carcinoma preparations as well as in the sera of many ovarian carcinoma patients. The occurrence of this antigen in patient sera has permitted the preparation of a competitive inhibition assay of ELISA signal using Mab CF511. The results of the ELISA inhibition of clinical specimens in this study indicated that the circulating CF511 antigen was elevated in the sera of approximately 70% of patients with common epithelial ovarian carcinoma. It was also apparent that the levels of the antigen in carcinoma patients were higher than those in healthy individuals or patients with benign diseases. Furthermore, higher antigen levels were found in the sera from ovarian carcinoma patients with advanced clinical stage (FIGO III and IV) than from those with the early stages of I or II. These data suggest that the circulating CF511 antigen levels may reflect the size of the tumour burden in ovarian carcinoma patients. On the other hand, elevated antigen levels were detected in sera from all patients with lung carcinoma and with breast carcinoma tested. These findings indicate that the detection of an elevated CF511 antigen level will be less helpful in distinguishing patients with ovarian carcinoma from those with either lung or breast carcinoma. The arbitarily chosen cut-off values of $>34.0\%$ ELISA inhibition (mean + 2 s.d.) resulted in a specificity of $<3.8\%$ false positive and a sensitivity of $>69.4\%$ true positives. The results also demonstrate that

**Table IV** Cross-reactivity of Mab CF511 was screened with other well recognised tumour marker antigens

| | *% inhibition of ELISA in various Mab CF511 concentrations* ($\mu g\ ml^{-1}$)[a] | | |
|---|---|---|---|
| *Tumour marker antigens* | *0.5* | *2.0* | *5.0* |
| CA19-9 | 0 | 0 | 0 |
| CA15-3 | 0 | 0 | 0 |
| CA125 | 0 | 0 | 0 |
| DU-PAN-2 | 0 | 0 | 0 |
| SLX | 0 | 0 | 0 |
| CSLEX | 0 | 0 | 0 |
| CF511 antigen | 100 | 100 | 100 |
| normal mouse serum | 0 | 0 | 0 |

[a]Mab CF511 diluted by 1% BSA in TBS was pre-incubated with standard antigens, CA 19-9 (240 U ml$^{-1}$), CA 15-3 (500 U ml$^{-1}$), CA125 (500 U ml$^{-1}$), DU-PAN-2 (1000 U ml$^{-1}$), SLX (360 U ml$^{-1}$) and CSLEX (224 U ml$^{-1}$), induced in the commercially available immunoassay kits. CF511 antigen (HACCM(-)) and normal mouse serum were used as negative and positive controls, respectively. Then the incubation mixtures were further incubated in antigen-coated microtitre wells and ELISA inhibition was estimated as described in Materials and methods.

CF511 antigen levels are not significantly elevated in patients with benign ovarian diseases or malignant ovarian tumours derived from germ cell or from mesenchymal cells and uterine fibroids with or without endometriosis. Similarly, patients with gastric, hepatic and colorectal carcinomas had no detectable elevation of circulating CF511 antigen. Thus, although an increased level of circulating CF511 antigen has been detected in certain other malignancies, the pattern of specificity suggests that measuring CF511 antigen levels may be useful for diagnosis of ovarian carcinoma and in terms of monitoring clinical course of ovarian epithelial carcinoma.

The CF511 antigen is a high molecular weight glycoprotein of 600 kDa. Enzymatic digestions or reagents treatments of CF511 antigen revealed that the antigen was partially sensitive to neuraminidase or proteinase K and quite sensitive to alkaline-borohydride but was resistant to trypsin or heat. Furthermore, periodate oxidation of the CF511 antigen reduced the electrophoretic mobility of CF511 antigen, but increased the reactivity against antibody in SDSPAGE-immunoblotting analysis as well as antigen coated ELISA. The increased antibody binding upon periodate oxidation suggests that epitope itself may not involve carbohydrate but involve exposed protein backbone which is revealed by the treatment. Since neuraminidase treatment of the antigen is able to reduce antibody binding, a contribution by the terminal sialic acid to the epitope cannot be ruled out, that is, the sialylated carbohydrate chain is not a direct component of the antigenic determinant but responsible for maintaining of its conformation. Furthermore, the finding of sensibility to alkaline-borohydride treatment suggests the serine-, threonine-type carbohydrate chains are also required for maintaining antigenicity. The reduced mobility of CF511 antigen in SDSPAGE was noted not only in the neuraminidase treatment but also in the periodate oxidation. The former is mainly because of the abolition of the negative charge by desialylation of the sialylated glycoprotein where the high levels of sialic acid produce a significant negative charge on the SDS-glycoprotein complex. This curious phenomenon has been reported in other proteins, including high molecular weight tumour-associated glycoproteins (Gahmberg & Anderson, 1982; Johnson *et al.*, 1986; Bray *et al.*, 1987). The latter is probably related to the conformational change of the antigen molecule due to cleavage of carbohydrate vicinal groups without altering the backbone-peptide chains (Sekine *et al.*, 1985). These findings suggest that the presence of several sialyl carbohydrate chains on a peptide backbone is probably required for maintaining CF511 antigenicity. However, it is difficult to ascertain the exact molecular properties of CF511 antigen from the data obtained from the treatment of non-purified antigen. Purification of the antigen is currently under investigation.

Although may Mabs generated against ovarian carcinoma have been described, the possibility for sero-diagnosis of ovarian carcinoma is demonstrated in only one Mab reported (Bast *et al.*, 1981, 1983). The most useful marker for patients with common epithelial ovarian carcinoma has been developed as CA125, a glycoprotein of high molecular weight (over 200 kDa) (O'Brien *et al.*, 1986; Masuho *et al.*, 1984; Matsuoka *et al.*, 1987) and probably in the range of $2-3 \times 10^3$ kDa by gel filtration determination (Davis *et al.*, 1986) in nature by Mab OC125 raised against ovarian cancer cell line. The CA125 antigenic determinant was recognised as periodate-, neuraminidase-resistant and protease-, heat-sensitive. These data strongly suggest that the CA125 antigenic determinant is composed, at least in part, of conformationally dependent peptide and is different from those of CF511 antigen. Bast *et al.* (1983) reported that elevated CA125 antigen levels could be detected in serum of more than 80% of patients with common epithelial ovarian carcinomas and that levels correlated with progression or regression of the diseases. However, CA125 has been found to be expressed not only in epithelial ovarian neoplasms but also in the derivatives of the coelomic epithelium, such as the muellerian epithelium, the linging cells from the coelomic cavity and lung tissues (O'Brien *et al.*, 1986; Kabawat *et al.*, 1983; Masuho *et al.*, 1984; Matsuoka *et al.*, 1987; Nouwen *et al.*, 1986). Serum levels of CA125 are therefore increased in a number of non-ovarian carcinoma patients including those with lung carcinoma and in some benign chronic pathologies (Nouwen *et al.*, 1986; Niloff *et al.*, 1984). In immunohistological analysis, CF511 antigen was present in common epithelial ovarian carcinoma (serous (100%), clear cell (100%), endometrioid (100%), and undifferentiated carcinoma (100%), mucinous (50%)) as well as in normal lung, breast, fallopian tube, uterus and thyroid gland. Serum levels of CF511 antigen are significantly elevated not only in ovarian carcinoma patients but also in either breast carcinoma or lung carcinoma patients regardless of the histological patterns. In contrast, no remarkable elevation of antigen levels was noted in patients with gastric carcinoma or with hepatoma and colorectal carcinoma. Furthermore, the incidence of false-positive reaction of CF511 antigen in patients with endometriosis was very much lower (22%) than that for the CA125 detection system (Niloff *et al.*, 1984). These results clearly indicate that CF511 antigen is distinct from CA125 and may be a generally useful marker for diagnosis and monitoring diseases in patients with common epithelial carcinoma as well as lung cell carcinoma and breast carcinoma.

It has been reported that some Mabs, originated against carcinomas derived from other organs, also cross-reacted with human ovarian carcinomas (Thor *et al.*, 1986; Friedman *et al.*, 1986). The most useful serodiagnostic Mab among these is known as DF3, and is prepared against a human breast carcinoma (Hayes *et al.*, 1985). However, two distinct differences in molecular characteristics between CF511 antigen and DF3 antigen are noticeable. The circulating CF511 antigen is highly detectable (10/10, 100%) in sera from patients with lung carcinoma, regardless of histology, whereas only 1/11 (9.1%) patients with lung carcinoma had DF3 positively detectable antigen levels (Friedman *et al.*, 1986). Another obvious difference between CF511 antigen and DF3 antigen is found in molecular weight determination using SDSPAGE-immunoblot analysis and/or gel filtration. DF3 identified a glycoprotein with a heterogenous molecular weight ranging from 300 to 450 kDa (Friedman *et al.*, 1986; Hayes *et al.*, 1985; Sekine *et al.*, 1985), whereas Mab CF511 recognises a band corresponding to a molecular weight of more than 600 kDa glycoprotein. Furthermore, by using competitive inhibition assay, the Mab CF511 and some antibodies against well characterised tumour marker antigens (CA19-9, CA125, CA15-3, DU-PAN-2, SLX, CSLEX) clearly react with different antigen epitope. These results suggest that the CF511 antigen does not seem to be related to previously characterised antigens.

Although Mab CF511 was prepared using fetal tissue extract from early first trimester as an immunogen followed by booster injection using human ovarian cancer cell line, HAC-2, the raising antigen, is not specific for fetal tissues and ovarian carcinomas, but is present on a few normal adult tissues and their derived carcinomas. Additionally, the antigen contents of various tissue extracts from normal adult are much higher than in those from fetus, as determined by antigen coated-ELISA as well as immunoblot analysis. Particularly, the antigen content in lung tissue is the same as that of common epithelial ovarian carcinoma. Despite the restricted distribution in some organs from normal adult, CF511 antigen circulates at significantly lower levels in normal individuals than in patients with carcinoma. These results suggest that the antigen is a normal tissue antigen that is anomalously shed or secreted into the circulation of epithelial ovarian carcinoma, lung carcinoma and breast carcinoma patients.

Although the initial purpose of obtaining a Mab directed towards onco-developmental antigens was not accomplished in this investigation, the strategy used here may be useful in the generation of new antibodies againsts onco-developmental antigens as well as cancer associated antigens. Such antibodies may be useful as tumour markers.

This work was supported by Grant-in-Aid for Cancer Research from the Ministry of Education, Science and Culture, Japan. The authors greatfully acknowledge the fine technical assistance of Miss K. Endoh and Miss K. Imamatsu, as well as the discussion and helpful suggestions of Dr Makoto Matsuda.

### References

BAST, R.C. Jr, FEENEY, M., LAZARUS, H., NADLER, L.M., COLVIN, R.B. & KNAPP, R.C. (1981). Reactivity of a monoclonal antibody with human ovarian carcinoma. *J. Clin. Invest.*, **68**, 1331.

BAST, R.C., Jr, KLUG, T.L., JENISON, E.J.R.N.E. & 8 others (1983). A radioimmunoassay using a monoclonal antibody to monitor the course of epithelial ovarian cancer. *N. Engl. J. Med.*, **309**, 883.

BHATTACHARYA, N., CHATTERJEE, S.K., BARLOW, J.J. & FUJI, H. (1982). Monoclonal antibodies recognizing tumor-associated antigen of human ovarian mucinous cystadenocarcinomas. *Cancer Res.*, **42**, 1650.

BRAY, K.R., KODA, J.E. & GAUR, P.K. (1987). Serum levels and biochemical characteristics of cancer-associated antigen CA-549, a circulating breast cancer marker. *Cancer Res.*, **47**, 5853.

CROGHAN, G.A., WINGATE, M.B., CAMARRA, M. & 6 others (1984). Reactivity of monoclonal antibody F36/22 with human ovarian adenocarcinomas. *Cancer Res.*, **44**, 1954.

DAVIS, H.M., ZURAWSKI, V.R. Jr, BAST, R.C., Jr, & KLUG, T.L. (1986). Characterization of the CA125 antigen associated with human epithelial ovarian carcinomas. *Cancer Res.*, **46**, 6143.

FEIZI, T. (1985). Demonstration by monoclonal antibodies that carbohydrate structures of glycoproteins and glycolipids are onco-developmental antigens. *Nature*, **314**, 53.

FRIEDMAN, E.L., HAYES, D.F. & KUFE, D.W. (1986). Reactivity of monoclonal antibody DF3 with a high molecular weight antigen expressed in human ovarian carcinomas. *Cancer Res.*, **46**, 5189.

FUKUSHI, Y., NUDELMAN, E., LEVERY, S.B., HAKOMORI, S. & RAUVALA, H. (1984). Novel fucolipids accumulating in human adenocarcinoma, III. A hybridoma antibody (FH6) defining a human cancer-associated difucoganglioside ($VI^3NeuAcV^3III^3Fuc_2nLc_6$). *J. Biol. Chem.*, **259**, 10511.

FUKUSHIMA, K., HIROTA, M., TERASAKI, P.I. & 7 others (1984). Characterization of sialosylated Lewis$^x$ as a new tumor-associated antigen. *Cancer Res.*, **44**, 5279.

GAHMBERG, C. & ANDERSON, L. (1982). Role of sialic acid in the mobility of membrane proteines containing O-linked oligosaccharides on polyacrylamide gel electrophoresis in sodium dodecyl sulfate. *Eur. J. Biochem.*, **122**, 581.

HAYES, D.F., SEKINE, H., OHNO, T., ABE, M., KEEFE, K. & KUFE, D.W. (1985). Use of a murine monoclonal antibody for detection of circulating plasma DF3 antigen levels in breast cancer patients. *J. Clin. Invest.*, **75**, 1671.

JOHNSON, V.G., SCHLOM, J., PATERSON, A.J., BENNET, J., MAGNANI, J.L. & COLCHER, D. (1986). Analysis of a human tumor-associated glycoprotein (TAG-72) identified by monoclonal antibody B72.3. *Cancer Res.*, **46**, 850.

KABAWAT, S.E., BAST, R.C. Jr, BHAN, A.K., WELCH, W.R., KNAPP, R.C. & COLVIN, R.B. (1983). Tissue distribution of a coelomic-epithelium-related antigen recognized by the monoclonal antibody OC125. *Int. J. Gynecol. Pathol.*, **2**, 275.

KOPROWSKI, H., STEPLEWSKI, Z., MITCHELL, K., HERLYN, M., HERLYN, D. & FUHRER, P. (1979). Colorectal carcinoma antigens detected by hybridoma antibodies. *Somat. Cell Genet.*, **5**, 957.

LOWRY, O.H., ROSEBROUGH, N.J., FARR, A.L. & RANDALL, R.J. (1951). Protein measurement with the Folin phenol reagent. *J. Biol. Chem.*, **193**, 265.

MASUHO, Y., ZALTSKY, M., KNAPP, R.C. & BAST, R.C. Jr (1984). Interaction of monoclonal antibodies with cell surface antigens of human ovarian carcinomas. *Cancer Res.*, **44**, 2813.

MATSUOKA, Y., NAKASHIMA, T., ENDO, K. & 7 others (1987). Recognition of ovarian cancer antigen Ca125 by murine monoclonal antibody produced by immunization of lung cancer cells. *Cancer Res.*, **47**, 6335.

MATTES, M.J., CORDON-CARDO, C., LEWIS, J.L. Jr, OLD, L.J. & LLOYD, K.O. (1984). Cell surface antigens of human ovarian and endometrial carcinoma defined by monoclonal antibodies. *Proc. Natl Acad. Sci. USA*, **81**, 568.

MATTES, M.J., LOOK, K., FURUKAWA, K. & 4 others (1987). Mouse monoclonal antibodies to human epithelial differentiation antigens expressed on the surface of ovarian carcinoma ascites cells. *Cancer Res.*, **47**, 6741.

METZGAR, R.A., RODRIGUEZ, N., FINN, O.J. & 7 others (1984). Detection of a pancreatic cancer associated antigen (DU-PAN-2 antigen) in serum and ascites of patients with adenocarcinoma. *Proc. Natl Acad. Sci. USA*, **81**, 5242.

MIOTTI, S., AGUANNO, S., CANEVARI, S. & 4 others (1985). Biochemical analysis of human ovarian cancer-associated antigens defined by murine monoclonal antibodies. *Cancer Res.*, **45**, 826.

MIOTTI, S., CANEVARI, S., MEZZANZANICA, D. & 5 others (1987). Characterization of human ovarian carcinoma-associated antigens defined by novel monoclonal antibodies with tumor-restricted specificity. *Int. J. Cancer*, **39**, 297.

NAKABAYASHI, H., TAKETA, K., MIYANO, K., YAMANE, T. & SATO, J. (1982). Growth of human hepatoma cell lines with differentiated functions in chemically defined medium. *Cancer Res.*, **42**, 3858.

NAKAGAWA, S., TSUJI, Y., MASUDA, N., NISHIURA, H. & ISOJIMA, S. (1987). Establishment of a murine monoclonal antibody against human clear cell carcinoma and analysis of the corresponding antigen. *Gynecol. Oncol.*, **28**, 318.

NILOFF, J.M., KNAPP, R.C., SCHAETZL, E.M., REYNOLDS, C. & BAST, R.C. Jr (1984). CA125 antigen levels in obstetrics and gynecologic patients. *Obstet. Gynecol.*, **64**, 703.

NOUWEN, E.J., POLLET, D.E., EERDEKENS, M.W., HENDRIX, P.G., BRIERS, T.W. & DE BROE, M.E. (1986). Immunohistochemical localization of placental alkaline phosphatase, carcinoembryonic antigen, and cancer antigen 125 in normal and neoplastic human lung. *Cancer Res.*, **46**, 866.

O'BRIEN, T.J., HARDIN, J.W., BANNON, G.A., NORRIS, J.S. & QUIRK, J.G. Jr (1986). Ca 125 antigen in human amniotic fluid and fetal membranes. *Am. J. Obstet. Gynecol.*, **155**, 50.

PETTERSSON, F. (1985). Annual report on the results of treatment in gynecological cancer. In *FIGO Annual Report, vol. 19*, Patterson, F. (ed.) p. 210. International Federation of Gynecology and Obstetrics: Stockholm.

SAKAKIBARA, K., UEDA, R., OHTA, M., NAKASHIMA, N., TOMODA, Y. & TAKAHASHI, T. (1988). Three novel mouse monoclonal antibodies, OM-A, OM-B, and OM-C, reactive with mucinous type ovarian tumors. *Cancer Res.*, **48**, 4639.

SEKINE, H., OHNO, T. & KUFE, D.W. (1985). Purification and characterization of a high molecular weight glycoprotein detectable in human milk and breast carcinomas. *J. Immunol.*, **135**, 3610.

TAGLIABUE, E., MENARD, S., TORRE, D.G. & 4 others (1985). Generation of monoclonal antibodies reacting with human epithelial ovarial cancer. *Cancer Res.*, **45**, 379.

THOR, A., GORSTEIN, F., OHUCHI, N., SZPAK, C.A., JOHNSTON, W.W. & SCHOLM, J. (1986). Tumor-associated glycoprotein (TAG-72) in ovarian carcinomas defined by monoclonal antibody B72.3. *J. Natl Cancer Inst.*, **76**, 995.

TOWBIN, H., STAEHELIN, T. & GORDON, J. (1979). Electrophoretic transfer of proteins from polyacrylamide gels to nitrocellulose sheets: procedure and some applications. *Proc. Natl Acad. Sci. USA*, **76**, 4350.

TSUJI, Y., SUZUKI, T., NISHIURA, H., TAKEMURA, T. & ISOJIMA, S. (1985). Identification of two different surface epitopes of human ovarian epithelial carcinomas by monoclonal antibodies. *Cancer Res.*, **45**, 2358.


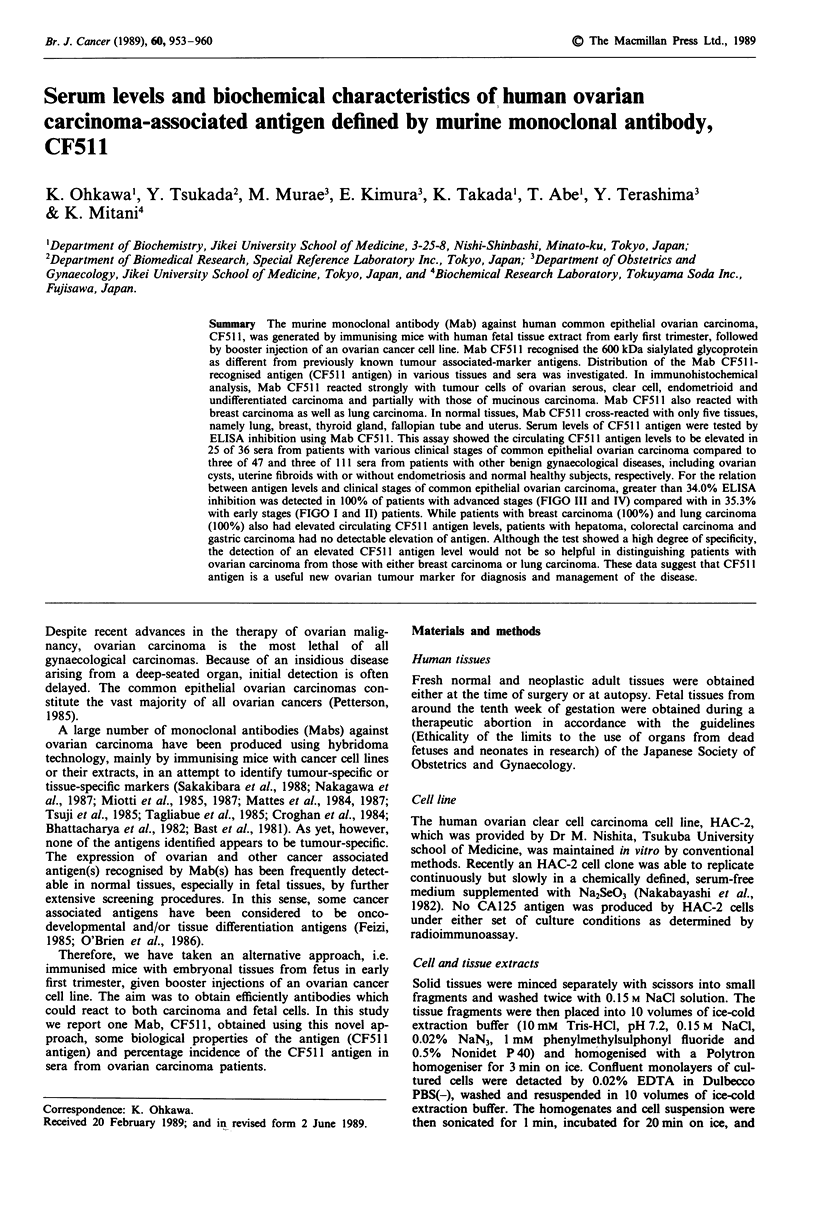

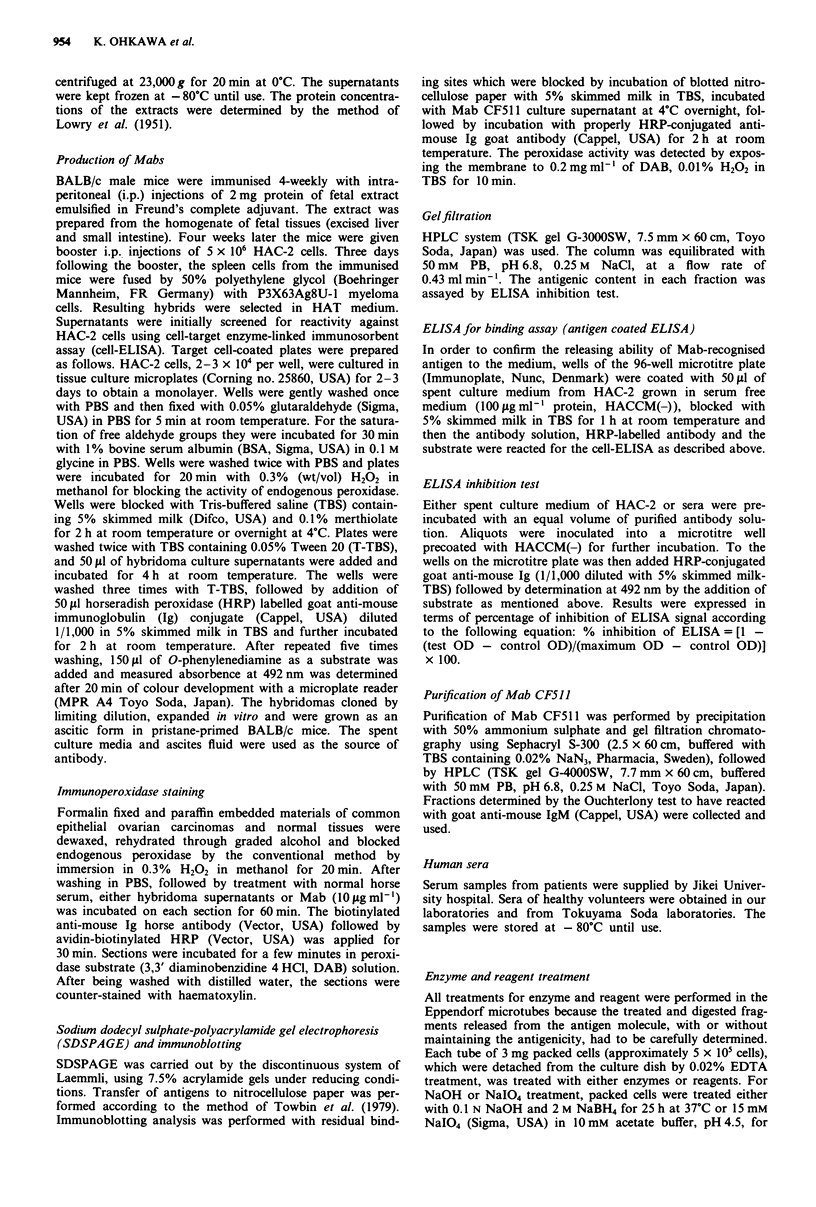

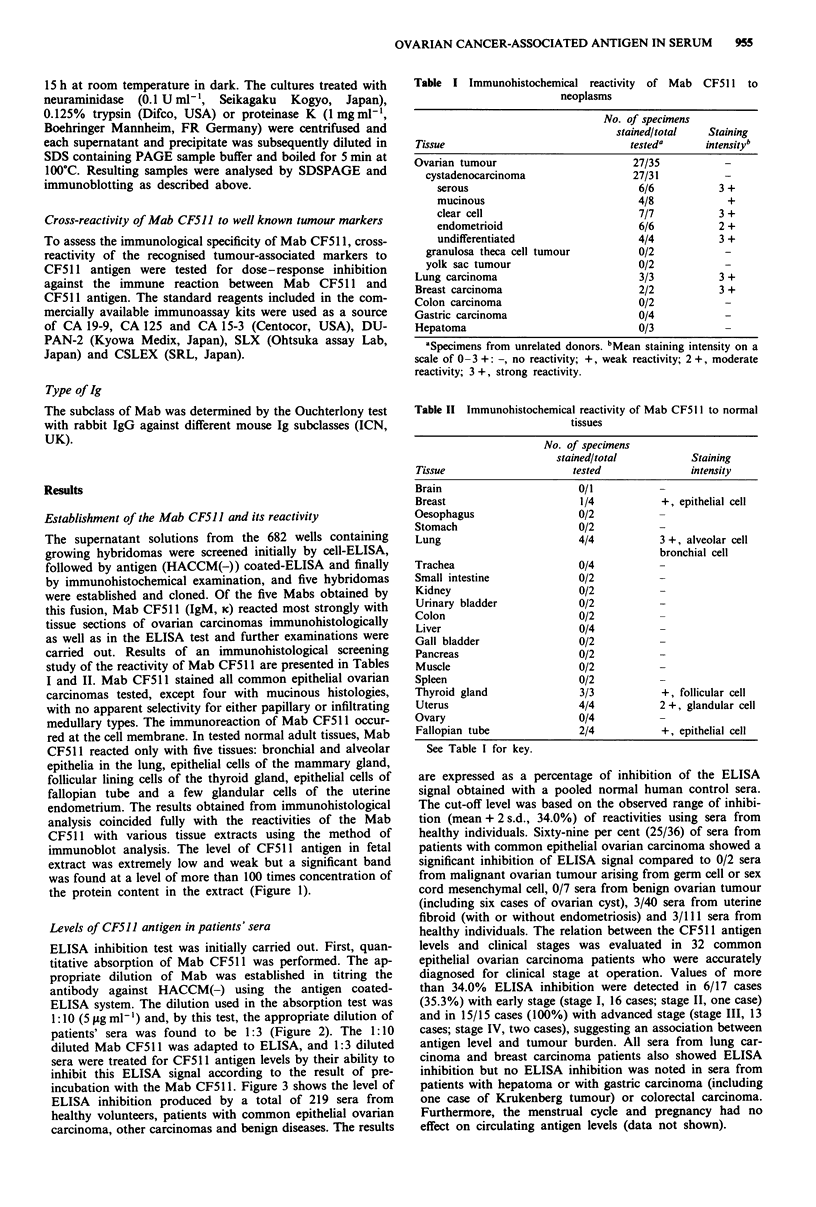

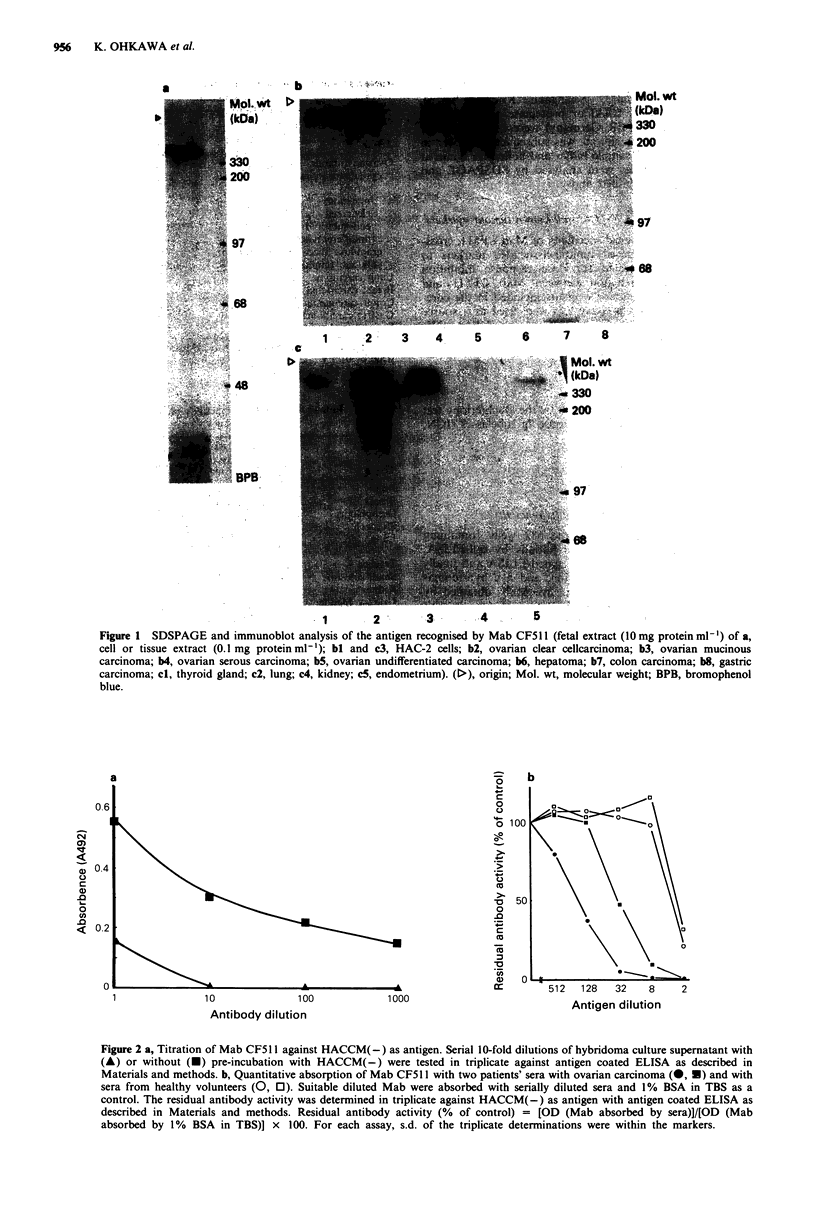

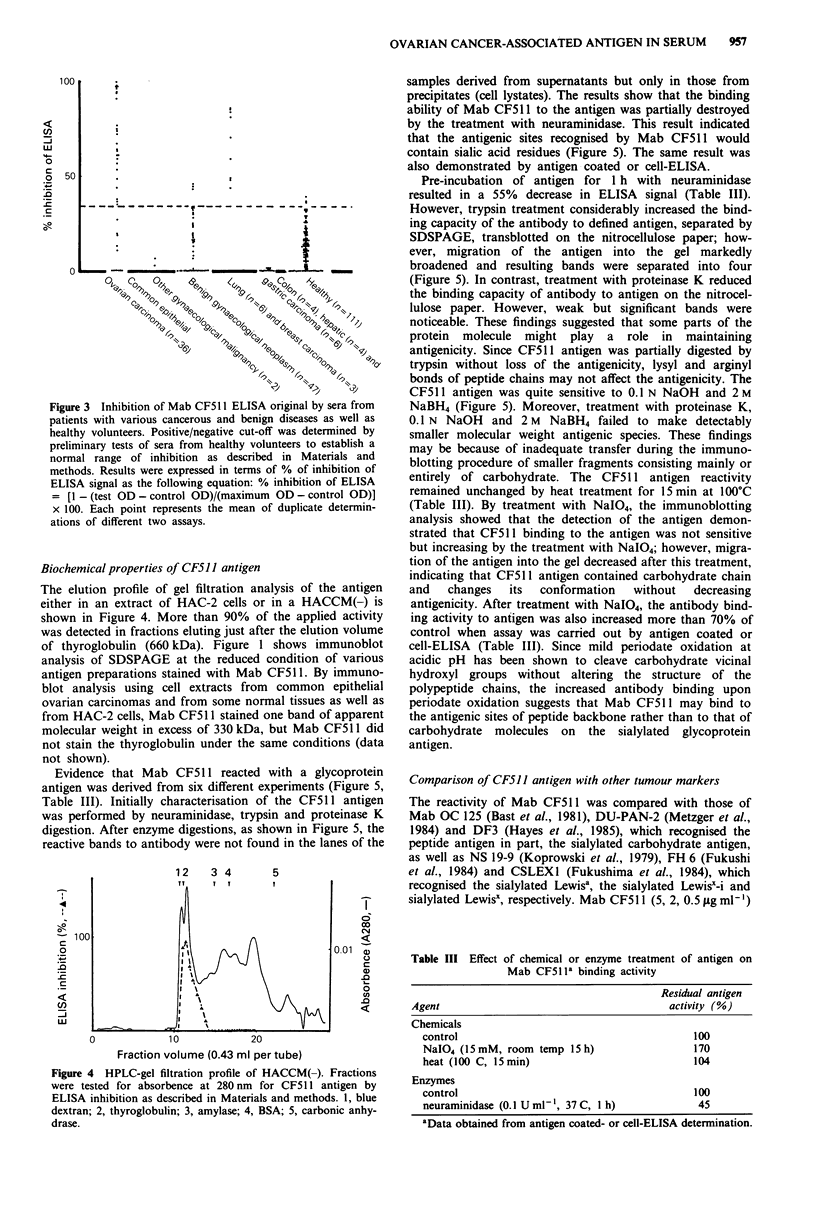

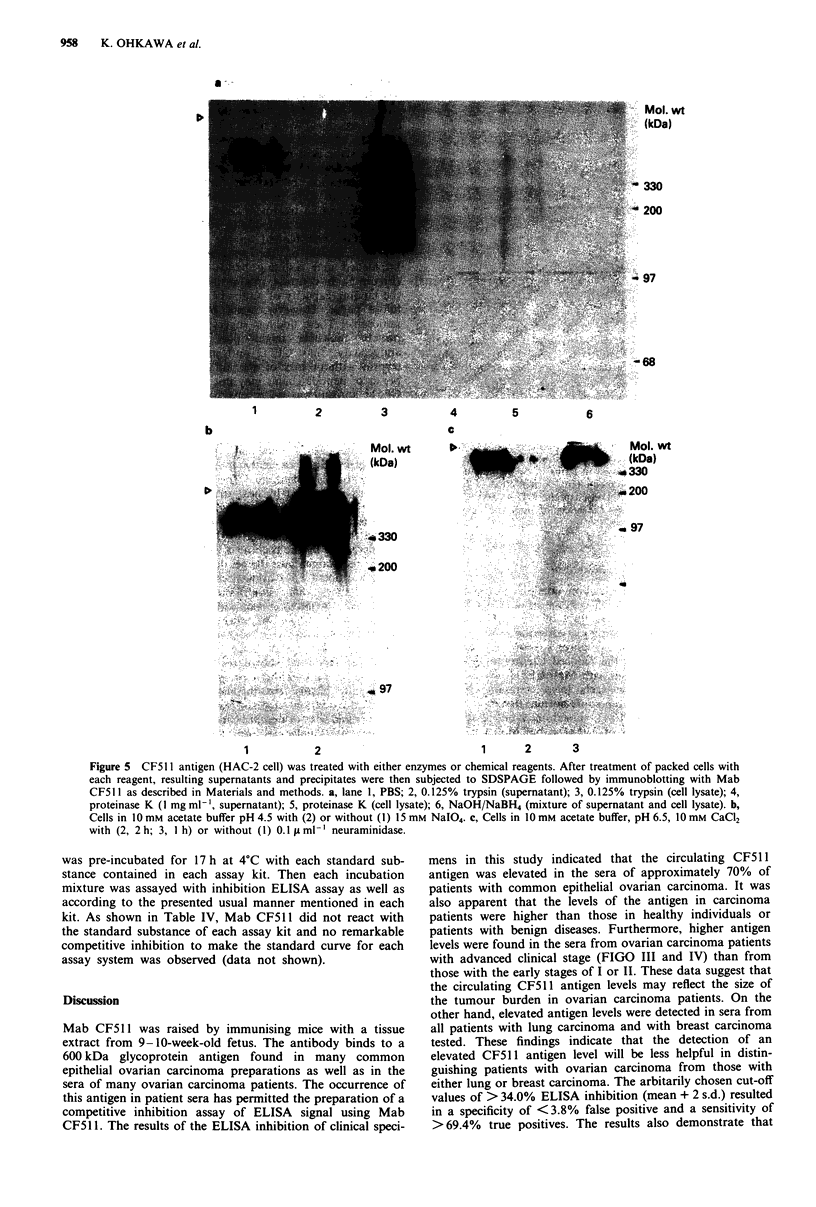

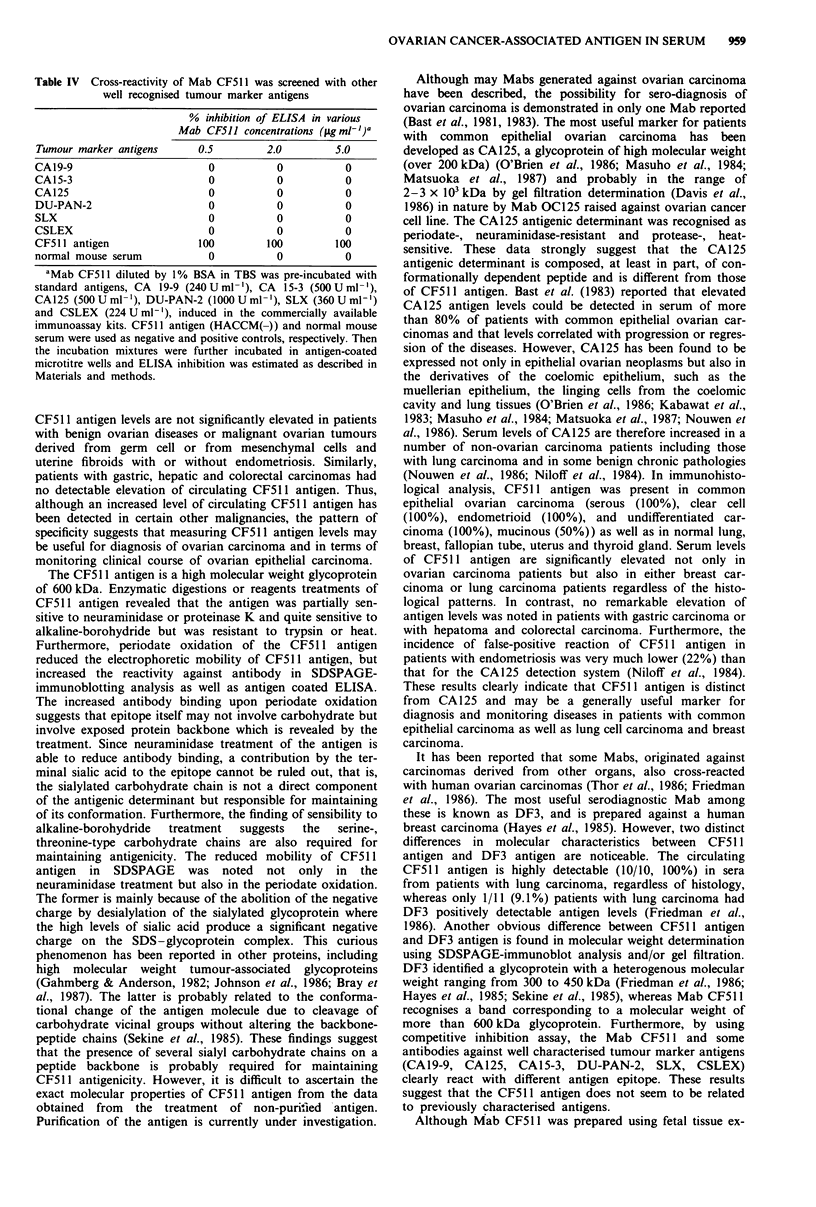

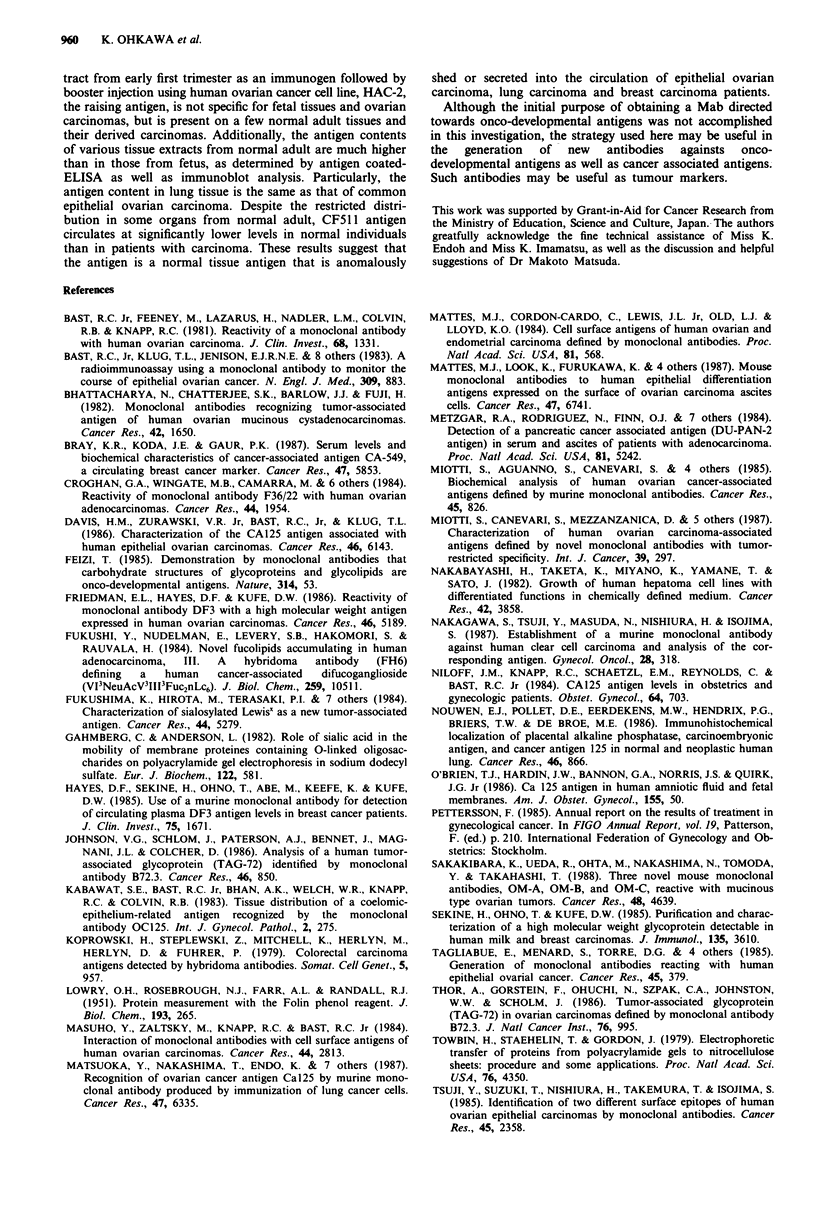


## References

[OCR_01006] Bast R. C., Feeney M., Lazarus H., Nadler L. M., Colvin R. B., Knapp R. C. (1981). Reactivity of a monoclonal antibody with human ovarian carcinoma.. J Clin Invest.

[OCR_01011] Bast R. C., Klug T. L., St John E., Jenison E., Niloff J. M., Lazarus H., Berkowitz R. S., Leavitt T., Griffiths C. T., Parker L. (1983). A radioimmunoassay using a monoclonal antibody to monitor the course of epithelial ovarian cancer.. N Engl J Med.

[OCR_01015] Bhattacharya M., Chatterjee S. K., Barlow J. J., Fuji H. (1982). Monoclonal antibodies recognizing tumor-associated antigen of human ovarian mucinous cystadenocarcinomas.. Cancer Res.

[OCR_01021] Bray K. R., Koda J. E., Gaur P. K. (1987). Serum levels and biochemical characteristics of cancer-associated antigen CA-549, a circulating breast cancer marker.. Cancer Res.

[OCR_01026] Croghan G. A., Wingate M. B., Gamarra M., Johnson E., Chu T. M., Allen H., Valenzuela L., Tsukada Y., Papsidero L. D. (1984). Reactivity of monoclonal antibody F36/22 with human ovarian adenocarcinomas.. Cancer Res.

[OCR_01031] Davis H. M., Zurawski V. R., Bast R. C., Klug T. L. (1986). Characterization of the CA 125 antigen associated with human epithelial ovarian carcinomas.. Cancer Res.

[OCR_01036] Feizi T. (1985). Demonstration by monoclonal antibodies that carbohydrate structures of glycoproteins and glycolipids are onco-developmental antigens.. Nature.

[OCR_01041] Friedman E. L., Hayes D. F., Kufe D. W. (1986). Reactivity of monoclonal antibody DF3 with a high molecular weight antigen expressed in human ovarian carcinomas.. Cancer Res.

[OCR_01045] Fukushi Y., Nudelman E., Levery S. B., Hakomori S., Rauvala H. (1984). Novel fucolipids accumulating in human adenocarcinoma. III. A hybridoma antibody (FH6) defining a human cancer-associated difucoganglioside (VI3NeuAcV3III3Fuc2nLc6).. J Biol Chem.

[OCR_01052] Fukushima K., Hirota M., Terasaki P. I., Wakisaka A., Togashi H., Chia D., Suyama N., Fukushi Y., Nudelman E., Hakomori S. (1984). Characterization of sialosylated Lewisx as a new tumor-associated antigen.. Cancer Res.

[OCR_01057] Gahmberg C. G., Andersson L. C. (1982). Role of sialic acid in the mobility of membrane proteins containing O-linked oligosaccharides on polyacrylamide gel electrophoresis in sodium dodecyl sulfate.. Eur J Biochem.

[OCR_01063] Hayes D. F., Sekine H., Ohno T., Abe M., Keefe K., Kufe D. W. (1985). Use of a murine monoclonal antibody for detection of circulating plasma DF3 antigen levels in breast cancer patients.. J Clin Invest.

[OCR_01071] Johnson V. G., Schlom J., Paterson A. J., Bennett J., Magnani J. L., Colcher D. (1986). Analysis of a human tumor-associated glycoprotein (TAG-72) identified by monoclonal antibody B72.3.. Cancer Res.

[OCR_01075] Kabawat S. E., Bast R. C., Bhan A. K., Welch W. R., Knapp R. C., Colvin R. B. (1983). Tissue distribution of a coelomic-epithelium-related antigen recognized by the monoclonal antibody OC125.. Int J Gynecol Pathol.

[OCR_01081] Koprowski H., Steplewski Z., Mitchell K., Herlyn M., Herlyn D., Fuhrer P. (1979). Colorectal carcinoma antigens detected by hybridoma antibodies.. Somatic Cell Genet.

[OCR_01087] LOWRY O. H., ROSEBROUGH N. J., FARR A. L., RANDALL R. J. (1951). Protein measurement with the Folin phenol reagent.. J Biol Chem.

[OCR_01092] Masuho Y., Zalutsky M., Knapp R. C., Bast R. C. (1984). Interaction of monoclonal antibodies with cell surface antigens of human ovarian carcinomas.. Cancer Res.

[OCR_01097] Matsuoka Y., Nakashima T., Endo K., Yoshida T., Kunimatsu M., Sakahara H., Koizumi M., Nakagawa T., Yamaguchi N., Torizuka K. (1987). Recognition of ovarian cancer antigen CA125 by murine monoclonal antibody produced by immunization of lung cancer cells.. Cancer Res.

[OCR_01103] Mattes M. J., Cordon-Cardo C., Lewis J. L., Old L. J., Lloyd K. O. (1984). Cell surface antigens of human ovarian and endometrial carcinoma defined by mouse monoclonal antibodies.. Proc Natl Acad Sci U S A.

[OCR_01109] Mattes M. J., Look K., Furukawa K., Pierce V. K., Old L. J., Lewis J. L., Lloyd K. O. (1987). Mouse monoclonal antibodies to human epithelial differentiation antigens expressed on the surface of ovarian carcinoma ascites cells.. Cancer Res.

[OCR_01115] Metzgar R. S., Rodriguez N., Finn O. J., Lan M. S., Daasch V. N., Fernsten P. D., Meyers W. C., Sindelar W. F., Sandler R. S., Seigler H. F. (1984). Detection of a pancreatic cancer-associated antigen (DU-PAN-2 antigen) in serum and ascites of patients with adenocarcinoma.. Proc Natl Acad Sci U S A.

[OCR_01121] Miotti S., Aguanno S., Canevari S., Diotti A., Orlandi R., Sonnino S., Colnaghi M. I. (1985). Biochemical analysis of human ovarian cancer-associated antigens defined by murine monoclonal antibodies.. Cancer Res.

[OCR_01127] Miotti S., Canevari S., Ménard S., Mezzanzanica D., Porro G., Pupa S. M., Regazzoni M., Tagliabue E., Colnaghi M. I. (1987). Characterization of human ovarian carcinoma-associated antigens defined by novel monoclonal antibodies with tumor-restricted specificity.. Int J Cancer.

[OCR_01133] Nakabayashi H., Taketa K., Miyano K., Yamane T., Sato J. (1982). Growth of human hepatoma cells lines with differentiated functions in chemically defined medium.. Cancer Res.

[OCR_01139] Nakagawa S., Tsuji Y., Masuda N., Nishiura H., Isojima S. (1987). Establishment of a murine monoclonal antibody against human clear cell carcinoma and analysis of the corresponding antigen.. Gynecol Oncol.

[OCR_01145] Niloff J. M., Knapp R. C., Schaetzl E., Reynolds C., Bast R. C. (1984). CA125 antigen levels in obstetric and gynecologic patients.. Obstet Gynecol.

[OCR_01150] Nouwen E. J., Pollet D. E., Eerdekens M. W., Hendrix P. G., Briers T. W., De Broe M. E. (1986). Immunohistochemical localization of placental alkaline phosphatase, carcinoembryonic antigen, and cancer antigen 125 in normal and neoplastic human lung.. Cancer Res.

[OCR_01157] O'Brien T. J., Hardin J. W., Bannon G. A., Norris J. S., Quirk J. G. (1986). CA 125 antigen in human amniotic fluid and fetal membranes.. Am J Obstet Gynecol.

[OCR_01168] Sakakibara K., Ueda R., Ohta M., Nakashima N., Tomoda Y., Takahashi T. (1988). Three novel mouse monoclonal antibodies, OM-A, OM-B, and OM-C, reactive with mucinous type ovarian tumors.. Cancer Res.

[OCR_01174] Sekine H., Ohno T., Kufe D. W. (1985). Purification and characterization of a high molecular weight glycoprotein detectable in human milk and breast carcinomas.. J Immunol.

[OCR_01179] Tagliabue E., Mènard S., Della Torre G., Barbanti P., Mariani-Costantini R., Porro G., Colnaghi M. I. (1985). Generation of monoclonal antibodies reacting with human epithelial ovarian cancer.. Cancer Res.

[OCR_01184] Thor A., Gorstein F., Ohuchi N., Szpak C. A., Johnston W. W., Schlom J. (1986). Tumor-associated glycoprotein (TAG-72) in ovarian carcinomas defined by monoclonal antibody B72.3.. J Natl Cancer Inst.

[OCR_01190] Towbin H., Staehelin T., Gordon J. (1979). Electrophoretic transfer of proteins from polyacrylamide gels to nitrocellulose sheets: procedure and some applications.. Proc Natl Acad Sci U S A.

[OCR_01196] Tsuji Y., Suzuki T., Nishiura H., Takemura T., Isojima S. (1985). Identification of two different surface epitopes of human ovarian epithelial carcinomas by monoclonal antibodies.. Cancer Res.

